# Is Imatinib Maintenance Required for Patients with Relapse Chronic Myeloid Leukemia Post-Transplantation Obtaining CMR? A Pilot Retrospective Investigation

**DOI:** 10.1371/journal.pone.0065981

**Published:** 2013-06-18

**Authors:** Hua Jin, Yiying Xiong, Jing Sun, Yu Zhang, Fen Huang, Hongsheng Zhou, Zhiping Fan, Dan Xu, Yongqiang Wei, Min Dai, Ru Feng, Qifa Liu

**Affiliations:** Department of Hematology, Nanfang Hospital, Southern Medical University, Guangzhou, China; B.C. Cancer Agency, Canada

## Abstract

Imatinib can induce complete molecular remission (CMR) in relapse chronic myelogenous leukemia (CML) after allogeneic hematopoietic stem cell transplantation, but it is indefinite whether imatinib is required to maintain CMR. We retrospectively reviewed 37 relapse CML post-transplants treated with imatinib (n = 20) or donor lymphocyte infusion (DLI) (n = 17). The rate of CMR was 85% and 76.47% (*P* = 0.509) and treatment-related mortality was 0% and 29.4% (*P* = 0.019), respectively, in imatinib and DLI groups. Fifteen patients obtaining CMR voluntarily ceased imatinib, and did not experience relapse. The 8-year overall survival (OS) after relapse was 85%±8% and 40.3±12.1% (*P* = 0.017), and disease-free survival (DFS) after relapse was 85%±8% and 40.3±12.1% (*P* = 0.011), respectively, in imatinib and DLI groups. Imatinib resulted in higher OS and DFS than that of DLI in relapse CML. Imatinib maintenance might not be required for patients with relapse CML post-transplants after they achieved full donor chimerism and CMR.

## Introduction

Tyrosine kinase inhibitors (TKIs), such as imatinib, have led to a dramatic change in the management of chronic myelogenous leukemia (CML) [1]. Mature data on the use of imatinib in newly diagnosed CML in chronic phase (CML-CP) have shown a complete cytogenetic remission (CCR) rate of 82%–87% [2], [3].Therefore, allogeneic hematopoietic stem cell transplantation (allo-HSCT) is rarely recommended to patients with CML in the first chronic phase (CML-CP1) as a front-line therapy in most developed countries [4]–[6]. However, the potential for TKIs to effect cures is a matter of discussion. Allo-HSCT remains the only established curative approach for CML, especially in patients with CML in an advanced phase (accelerated phase (AP) and blast phase (BP)), and those in whom TKIs therapy has failed [7], [8]. The overall survival (OS) after allo-HSCT is estimated to range from 40% to 80%, with relapse and graft versus host disease (GVHD) as the main causes of death[9]–[11]. Before the era of TKIs, the relapse CML post-transplants had been treated with several therapeutic strategies, including Interferon alpha (IFN-a) [12], [13], a second transplantation [14], and donor lymphocyte infusion (DLI) [15]. DLI can result in complete molecular remission (CMR) in a high proportion, thus making it as the standard front-line approach for the relapse CML post-transplants [16]–[18]. The disadvantage of DLI is associated with the development of severe GVHD and marrow aplasia, which are a major contributing factor of death [15], [19]. In addition, DLI is not an option because of unavailability of the original donor, and contraindicated in patients with pre-existing GVHD [20]. The potential life-threatening side effects and unavailability of DLI make the use of TKI a highly attractive treatment for the relapse CML post-transplants [21]–[23]. Some studies have documented that imatinib is capable of inducing CMR for the relapse CML post-transplants [21], [24], [25]. Compared with DLI therapy, there was a trend towards higher rates of OS in the imatinib therapy, because of lower treatment-related death such as GVHD and marrow aplasia [26], [27]. However, it is indefinite whether continued imatinib therapy is required to maintain this response for patient obtaining CMR. In this report, we retrospectively compared the efficacy between imatinib and DLI to the relapse CML post-transplants, and evaluated the results of ceasing imatinib in the patients who had achieved CMR and complete donor chimerism via imatinib treatment.

## Materials and Methods

### Patients and Transplants

Thirty-seven patients with relapse CML, who underwent allo-HSCT from May 1999 to August 2011, were enrolled in this retrospective analysis. There were 14 females and 23 males. Median age of patients was 38 (range 12–57) years at the time of transplants. The median interval from diagnosis to transplants was 14.8 (range 2.4–61.1) months. Twenty-three patients were in CP, 2 in AP, and 12 in BP before transplants. At the time of transplants, 6 patients were complete hematologic remission (CHR) and 2 patients were partial hematologic remission (PHR) in 14 patients with advanced phase. Thirteen of 33 patients received imatinib therapy before transplants, including 4 patients who were failure or intolerance to imatinib and 9 patients who were responsive to imatinib. Twenty-four patients received HLA-matched sibling and 8 received matched unrelated donor transplants. Four patients received HLA-mismatched related donor and one mismatched unrelated donor transplants. Two conditioning regimens were used [28]. Before March 2003, most patients received total-body irradiation (TBI) and cyclophosphamide (CY) conditioning regimen. After April 2003, most patients received modified BUCY conditioning regimen (busulfanCY cytarabine). All patients who were relapse in BP received TBI and CY conditioning regimen. And GVHD prophylaxis were performed according to our strategies previously described [29]. This study was performed in accordance with the modified Helsinki Declaration, and the protocol was approved by the Ethics Committee of Southern Medical University affiliated Nanfang Hospital before study initiation. All donors and recipients provided written informed consent themselves. And in the case of all minor participants, written informed consent was provided by a parent or guardian.

### Treatment Schedule in Relapse CML

Once leukemia relapse was diagnosed, immunosuppressants were tapered or discontinued if the patient’s condition was acceptable. Those who were in molecular or cytogenetic relapse tapered or discontinued immunosuppressants as a front-line therapy. If these patients were not responsive to this treatment after one month, DLI- or imatinib-based treatments were administrated. Those who were in molecular, cytogenetic or CP relapse received DLI or imatinib monotherapy as a front-line therapy, and the patients in advanced phase received DLI or imatinib combined with chemotherapy as a front-line therapy. The chemotherapy protocols included HA (Homoharringtonine+Cytarabine) or DA (Daunorubicin+Cytarabine). Of the 37 patients enrolled in this analysis, 17 patients accepted the DLI-based treatments and 20 accepted the imatinib-based treatments. All patients received the DLI-based treatments as a front-line therapy except for two patients who were not availability of the original donor and accepted the imatinib-based treatments before December 2004. After January 2005, all patients received the imatinib-based treatments as a front-line therapy except for 3 patients who accepted the DLI-based treatments because of the history of imatinib ineffectiveness.

In the DLI-based treatments, the patients relapsing in advanced phase received DLI therapy after a cycle of chemotherapy. Three patients all received DLI on a bulk-dose (1×10^8^ mononuclear cells/kg) regimen once every four weeks until patients obtained CCR or developed GVHD before 2001. After 2001, all patients received granulocyte colony stimulating factors (G-CSF) modified DLI according to an escalating-dose schedule. The schedule was that the initial mononuclear cell dose was 1×10^7^/kg for HLA-mismatched related and unrelated donor transplants and 2×10^7^/kg for HLA-matched sibling donor transplants. Briefly, the recipients with unrelated donor transplants received DLI in incremental doses of 1, 2, 4 and 8×10^7^ mononuclear cells/kg, and the doses of mononuclear cells/kg were incrementally 2, 4, 8 and 16×10^7^ for the recipients with HLA-matched sibling donor transplants every four weeks until they obtained CCR or developed GVHD. In the imatinib-based treatments, the patients relapsing in BP received the imatinib combined with chemotherapy. Generally, chemotherapy was not used after the patients achieved a CHR or received 2 cycles of chemotherapy. Imatinib at a dose of 400 mg/day was used in the patients with molecular, cytogenetic or CP relapse, and a dose of 600–800 mg/day was used in the patients with advanced phase relapse by the attending physician. If the patients with molecular or cytogenetic relapse did not achieve major molecular remission (MMR) or partial cytogenetic remission (PCyR) or the patients with CP relapse did not achieve PHR after one month of imatinib treatment, imatinib dose would be increased to 600 to 800 mg/day by the attending physician. After one month, if the patients were not responsive to these treatments, DLI would be used. And if the patients in advanced phase did not achieve a PHR after one month of imatinib treatment, DLI would be used. When the patients had recovered donor complete chimerism and had achieved CMR as defined by negative quantitative RQ-PCR at three consecutive time points within a period of 3 months, the patients continued or ceased imatinib therapy according to their willingness.

### Philadelphia Chromosome (Ph), BCR-ABL Fusion Gene and Chimerism Detecting

Cytogenetic study was assessed by fluorescence in situ hybridization (FISH). Molecular analyses were accomplished by polymerase chain reaction (PCR). And chimerism statuses of donor and recipient were analyzed by FISH in sex mismatched transplants or by short tandem repeat (STR) analysis in sex matched transplants every 1 month after treatment. After 3 months, the detection was done by every 2 months for one year, and subsequently every 3 months for 3 years after relapse. Before 2003, BCR-ABL mRNA was analyzed by nested PCR. Result was expressed as positive or negative only. After 2003, BCR-ABL mRNA was monitored by real-time quantitative PCR (RQ-PCR). Our method of RQ-PCR has a sensitivity of up to 1 in 10^5^ cells.

### Definition

The diagnosis criteria of CML includes CP, AP and BP according to literature [21], [30]. There are three different types of responses in CML: (1) a hematological response, (2) a cytogenetic response and (3) a molecular responses. Hematologic responses were classified as CHR, PHR and ineffectiveness (NR) [31], [32]. Cytogenetic responses were classified as CCR, PCyR, and no cytogenetic response. Molecular responses were classified as CMR, Major molecular response (MMR) and no molecular response [31]. The threshold for CMR was based on the level below which bcr-abl transcripts were no longer detectable (more than 4.5 log reduction from the averaged baseline level) [21]. CMR was confirmed by PCR analysis at three consecutive time points within a period of 3 months. Complete chimerism (CC) was defined as >95% donor cells detected; mixed chimerism (MC), as 5% to 95% donor cells detected [32]. The criteria of relapse includes Hematologic relapse, Cytogenetic relapse, and Molecular relapse according to literature [21].

### Statistical Analysis

Chi-squared, Mann-Whitney analyses were performed when applicable to analyze differences between cohorts of patients. Differences were statistically significant when two-sided *P* values were less than 0.05. The probabilities of OS and disease-free survival (DFS) were calculated using the methods of Kaplan and Meier.

## Results

### Patient Characteristics

Of the 37 patients enrolled in the analysis, relapse was hematologic in 28 cases (14 cases in CP, 4 in AP and 10 in BP) and cytogenetics in 9 cases. The median interval from transplants to relapse was 12.9 (range 2.2–30.3) months. The median donor chimerism at relapse was 61% (range 47%–89%) in sex-mismatched transplantation, which was analyzed by FISH. And the median donor chimerism at relapse was 72% (range 27%–89%) in sex-matched transplantation, which was analyzed by STR. FISH revealed that the median percentage of Philadelphia chromosome positive cells at relapse was 55% (range 39%–92%). Eleven patients were receiving immunosuppressant therapy at relapse, including 9 patients received immunosuppressants for GVHD prophylaxis and 2 patients for GVHD treatment. Based on initial treatments at the time of relapse, the patients were divided into DLI (n = 17) and imatinib (n = 20) groups. The characteristics of patients and transplants are summarized in Table 1. The characteristics of patients and transplants were not statistically different between the two groups.

**Table 1:** Characteristics of patients and transplants.

	Imatinib (n = 20)	DLI (n = 17)	*P*-value
Male/Female	11/9	12/5	0.330
Median age(years, range)	36 (12–57)	39 (26–50)	0.614
Median time from diagnosis to transplant (months)	15.5 (2.4–61.1)	11.7 (3.7–41.9)	0.34 5
Status of disease at transplants (CP/AP/BP)	13/1/6	10/1/6	0.707
Median time from transplant to relapse (months)	11.4 (2.6–22.2)	13.3 (2.2–30.3)	0.626
Disease status at relapse CytogeneticHematologic(CP/AP/BP)	7 7/2/4	2 7/2/6	0.121
Donor Chimerism at relapse (%)	72 (27–89)	61 (47–83)	0.123
Ph-positive cells at relapse (%)	55(39–91)	60(39–92)	0.831
GVHD before relapseYes/No	2/18	0/17	0.180
Immunosuppressats at relapse (prophylaxis/treatment)	4/2	5/0	0.969

DLI = donor lymphocyte infusion, CP = chronic phase, AP = accelerated phase, BP = blast phase, Ph = Philadelphia chromosome, GVHD = graft versus host disease.

### Treatment and Responses

Of the 17 patients receiving the DLI-based treatments, 9 patients received DLI monotherapy and 8 patients received DLI combined with chemotherapy. The median number of infusions per patient was 2 (range 1–4) doses. The median CD3+ cells was 5.75×10^7^/kg (range 3.15–6.5). After DLI-based treatments, 13 patients achieved CMR and 4 patients were not responsive to DLI-based treatments. Those 4 patients all died of leukemia progressing. The time to achieve CMR was a median 10 (range 2–18) months after DLI-based treatments.

Of the 20 patients receiving the imatinib-based treatments, 16 patients received imatinib monotherapy and 4 patients received imatinib combined with chemotherapy. After imatinib-based treatments, 17 patients achieved CMR and 3 patients did not obtain CMR, who were all relapse in BP. Of the 3 patients who did not obtain CMR, one patient obtained CHR and PCyR after 2 months imatinib treatment combined with one cycle of chemotherapy, but this patient occurred central nervous system (CNS) leukemia at 86 days after treatments and died of CNS leukemia at 101 days after relapse. Other 2 patients who were relapse in BP did not obtain response in one month after one cycle of chemotherapy and imatinib. Thus DLI was added in the two patients. Eventually, the 2 patients died of leukemia progressing at 44 days and 88 days after relapse, respectively. The 2 patients who were not responsive to imatinib did not have BCR-ABL1 kinase domain (KD) mutations via mutational analysis. The time to achieve CMR was a median 4 (range 2–11) months after imatinib-based treatments. The rate of CMR was not different statistically between imatinib and DLI groups (85% vs 76.47%, *P* = 0.509).

### Donor Chimerism

**Figure 1:**
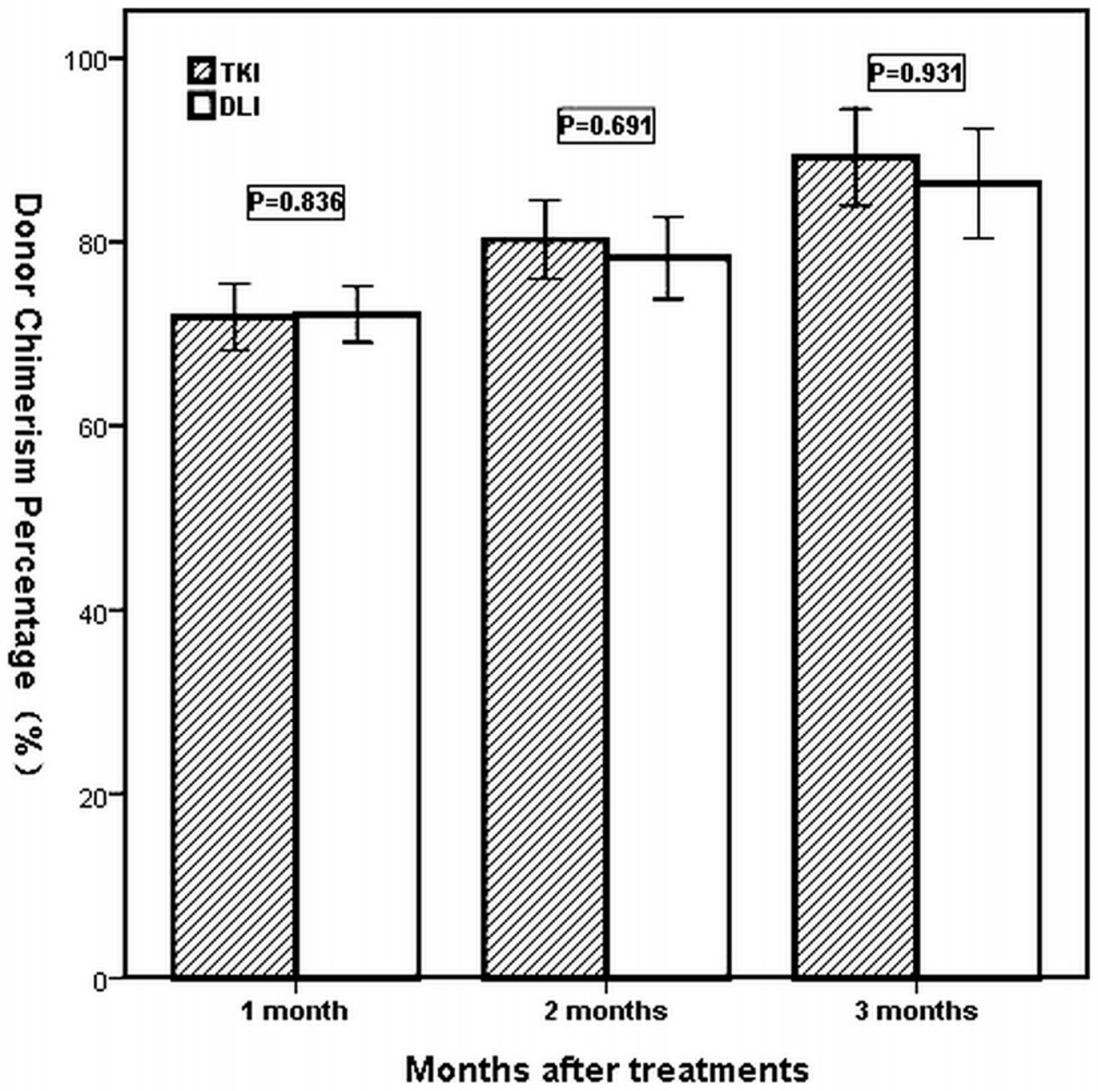
Donor chimerism in imatinib and DLI groups in 1, 2 and 3 months after treatments (*P* = 0.836, *P* = 0.691 and *P* = 0.931). The median donor chimerism in imatinib and DLI groups was 73% (range 27%–90%) vs 74% (range 47%–89%), 84% (range 11%–95%) vs 84% (range 28%–94%), and 96% (range 0%–100%) vs 97% (range 23%–100%), respectively, in 1, 2 and 3 months after treatments.

Chimerism analysis indicated that the median donor chimerism was 73% (range 27%–90%), 84% (range 11%–95%), and 97% (range 0%–100%), respectively, in 1, 2 and 3 months after treatments. In 6 months after treatments, 30 patients who achieved CCR all recovered full donor chimerism. The proportion of donor chimerism was not different statistically between two groups in 1, 2 and 3 months after treatments (*P* = 0.836, *P* = 0.691,and *P* = 0.931).The results are shown in Figure 1.

### Side Effects and Treatment-related Mortality

Of the 17 patients receiving the DLI-based treatments as a front-line therapy, 4 patients developed acute GVHD (2 grade II and 2 grade III) and 2 patients developed extensive chronic GVHD. Four patients died of GVHD, including acute GVHD in 2 and chronic GVHD in 2. One patient died of marrow aplasia. The DLI-related mortality was 29.4%.

Of the 20 patients receiving the imatinib-based treatments as a front-line therapy, none discontinued imatinib because of intolerable adverse effects. None developed GVHD, except for one patient who received imatinib combined with DLI treatments developed acute GVHD, and 2 patients developed limited chronic GVHD after imatinib-based treatments. Two patients who had chronic GVHD (1 limited, 1 extensive) at the time of imatinib therapy obtained a complete remission after imatinib-based treatments. The imatinib-related side effects included edema(n = 9), nausea (n = 8), muscle cramps (n = 7), diarrhea (n = 7), fatigue (n = 6), skin rashes(n = 6), abdominal pain (n = 5), headache (n = 5), neutropenia(n = 7), thrombocytopenia(n = 6), elevated liver enzymes (n = 2). No patients died of imatinib-related side effects. The rate of treatment-related mortality was higher in the DLI group than in the imatinib group(29.4% vs 0%, *P* = 0.019).

### Relapse and Survival

**Figure 2:**
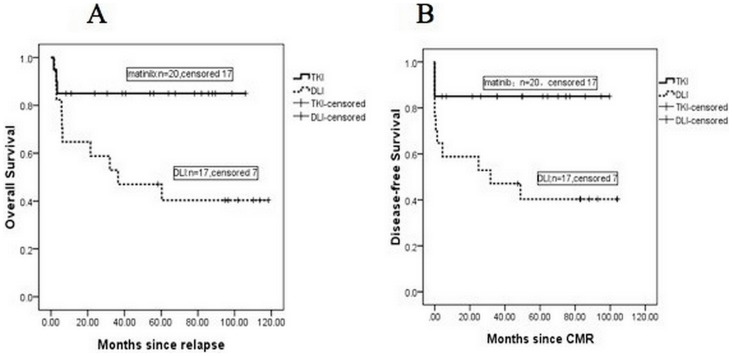
Overall Survival (A) and Disease-free Survival (B) in the imatinib and DLI groups. The 8-year overall survival (OS) after relapse was 85%±8% and 40.3±12.1% (*P* = 0.017), 8-year disease-free survival (DFS) after relapse was 85%±8% and 40.3±12.1% (*P* = 0.011), respectively, in the imatinib and DLI groups.

In the DLI group, with a median follow up of 36.5 (range 1.8–118.3) months after relapse, 7 patients were alive and 10 patients were dead. Causes of death included leukemia progressing (n = 3), GVHD (n = 4), marrow aplasia (n = 1), post-transplant lymphoproliferative disorder (n = 1), and second relapse (n = 1). In the imatinib group, with a median follow up of 54.7 (range 1.5–105.9) months after relapse, 17 patients were alive and 3 patients were dead. Causes of death included leukemia progressing (n = 3). The 8-year overall survival (OS) after relapse was 85%±8% and 40.3±12.1%(*P* = 0.017), 8-year disease-free survival (DFS) after relapse was 85%±8% and 40.3±12.1% (*P* = 0.011), respectively, in the imatinib and DLI groups. Both OS and DFS were higher in the imatinib group than in the DLI group (Figure 2A and 2B).

### Effect of Imatinib Cessation to Relapse

Fifteen of the 17 patients who recovered complete donor chimerisms and obtained CMR voluntarily ceased imatinib at a median 4 (range 3–15) months after CMR. With a median follow up of 59 (range 3–96) months after imatinib cessation, none of 15 patients experienced relapse. Of the two patients who are now maintained on imatinib, one was relapse in BP and the other one was relapse in AP. The period of imatinib treatment was 31 months and 8 months respectively after relapse.

## Discussion

Before the introduction of imatinib, DLI was the most frequently used therapeutic strategy for the relapse CML post-transplants [33], [34]. The response rate of DLI varies between 36 and 100% in relapse CML depending on disease stage at relapse [18], [24], [35], [36]. DLI-related mortality may be up to 20% [34], [37]. Some prospective and retrospective data have demonstrated that imatinib is capable of inducing durable molecular responses for relapse CML post-transplants [21], [24], [25], [36]. There are no abundant data on the comparison between imatinib and DLI treatment response rates and survival. Some reports have documented that the treatment response rate between imatinib and DLI was similar [24], [27], [38] and imatinib was superior to DLI in OS because of the lower treatment-related mortality in imatinib treatment [26], [27]. However, Weisser et al. [26] reported that the rate of CMR in imatinib-treated group was lower than that of DLI-treated group, and imatinib was inferior to DLI in DFS because of a higher relapse rate. In this report, our retrospective data also showed that the rate of CMR was not different between imatinib and DLI. But there was higher treatment-related mortality in the DLI treatment, thus imatinib was superior to DLI in OS and DFS. In our data, the DLI-related mortality (29.4%) was obviously higher than those in the literature [34], [37]. A reasonable interpretation of this result is that 3/4 patients who died of DLI-related complications received DLI on a bulk-dose, which results in a high incidence and mortality of GVHD and marrow aplasia [16]. In addition, we observed that 2 patients with chronic GVHD obtained a complete remission after imatinib-based treatments, which might be caused by imatinib regulating GVHD [39].

To date, whether the newly diagnosed CML who accepted imatinib as a front-line therapy and achieved CMR can safely cease imatinib therapy has been widely discussed [40]–[42]. Some reports had documented that the rate of relapse might be to up 60% after cessation of imatinib [43]–[45]. Therefore, in the guidelines of the National Comprehensive Cancer Network (NCCN), discontinuation of imatinib therapy is not recommended in the CML receiving imatinib as a front-line therapy and achieving CMR. Concerning the relapse CML after allo-HSCT, there are no large samples and prospective reports on whether imatinib can be discontinued after patients obtained CMR. Some case reports were in contradiction. Olavarria et al. [46] suggested that imatinib cessation was associated with high risk of relapse. Oppositely, sporadic case reports revealed that patients who ceased imatinib did not experience relapse [26], [47], [48]. As far as we know, our data had the largest samples about the cessation of imatinib treatment in the relapse CML patients of allo-HSCT. In this report, 15 of the 17 patients voluntarily ceased imatinib after they had achieved CMR for 3 months in full donor chimerism. With a median follow up of 59 months after imatinib cessation, no patients experienced molecular relapse. Reasonable interpretations of this phenomenon are that the leukemic burden of relapse CML post-transplant is less than newly diagnosed CML and there is graft-versus-leukemia (GVL) effect in the relapse CML of allo-HSCT. With imatinib support, minimal residual leukemia can be well controlled and a window of opportunity for GVL can be created [49]. Moreover, Wang et al. [50] reported that imatinib might enhance antigen presentation and overcome tumor-induced CD4+ T-cell tolerance, and thereby facilitate GVL. Based on these, we hypothesize that the reestablishment of donor complete chimerism and restoration of full GVL in the patient with relapse CML post-transplant make it feasible to discontinue imatinib and maintain the durable disease remission [25], [49]. The hypothesis needs to be confirmed in large samples and prospective studies. Furthermore, some studies found that a higher Sokal risk score was inversely associated with CCyR and MMR in imatinib first-line treatment [51], [52]. And Mahon et al. [45] reported that higher Sokal risk group had a higher molecular relapse after discontinuation of imatinib. In our study, patients with relapse CML post-transplantation who were in higher sokal risk group also had worse responses to imatinib. Taking into account of the limitations that only one patient who were relapse in AP ceased imatinib, whether patients with relapse CML post-transplantation and higher sokal score have a higher relapse rate after cessation of imatinib needs to be further discussed.

In addition, our data showed that the median interval from imatinib treatment to CMR was faster in the relapse CML post-transplants, compared with the newly diagnosed CML accepting imatinib as a front-line therapy [44]. The possible reason is that imatinib combined with GVL kill leukaemic cells [25].

### Conclusions

In conclusion, our data had documented that imatinib resulted in higher OS and DFS than that of DLI in the relapse CML post-transplants. Imatinib maintenance might not be required for patients with relapse CML post-transplants after they achieved full donor chimerism and CMR. Taking into account of the limitations that only low numbers of patients were studied, our results need to be confirmed in more patients and in prospective trials.
